# Fresh Frozen Homologous Rib Cartilage: A Narrative Review of a New Trend in Rhinoplasty

**DOI:** 10.3390/jcm13061715

**Published:** 2024-03-16

**Authors:** Giovanni Salzano, Giovanni Audino, Giovanni Dell’Aversana Orabona, Umberto Committeri, Stefania Troise, Antonio Arena, Luigi Angelo Vaira, Pietro De Luca, Alfonso Scarpa, Andrea Elefante, Antonio Romano, Luigi Califano, Pasquale Piombino

**Affiliations:** 1 Neurosciences Reproductive and Odontostomatological Sciences Department, University of Naples “Federico II”, 80131 Naples, Italy; giovannisalzanomd@gmail.com (G.S.); giovanni.dellaversanaorabona@unina.it (G.D.O.); umbertocommitteri@gmail.com (U.C.); stefy.troise@gmail.com (S.T.); dr.antonio.arena@gmail.com (A.A.); romano.antonio1972@gmail.com (A.R.); pasquale.piombino@gmail.com (P.P.); 2 Maxillofacial Surgery Operative Unit, Department of Medicine, Surgery and Pharmacy, University of Sassari, 07100 Sassari, Italy; luigi.vaira@gmail.com; 3 San Giovanni Addolorata Hospital, Otorhinolaryngology Unit, 00184 Roma, Italy; dr.dlp@hotmail.it; 4 Department of Medicine and Surgery, University of Salerno, Via Salvador Allende 43, 84081 Baronissi, Italy; alfonsoscarpa@yahoo.it; 5 Neuroradiology Unit, Department of Advance Biomedical Sciences, University of Naples “Federico II”, 80131 Naples, Italy; andrea.elefante@unina.it

**Keywords:** rhinoplasty, fresh frozen rib cartilage, rib cartilage graft, facial plastic surgery, dorsal augmentation

## Abstract

**Background**: Revision rhinoplasty is a technically demanding surgical procedure that can put every surgeon in trouble. The main issue of these cases is often an altered osteocartilaginous framework following over-resection during the first intervention. Moreover, the available septal or auricular cartilage for grafting is usually not enough. This review aims to examine contemporary advances in applications of fresh frozen cartilage in rhinoplasty. **Methods**: A structured review of the current literature (up to December 2023) was performed on four bibliographic databases: PubMed, EMBASE, Cochrane and Medline. The search terms were combinations of “Rhinoplasty” and “Cartilage Graft”, “Allograft” or “Fresh Frozen Cartilage”. The citations of selected studies and review articles were also evaluated if present. **Results**: The research resulted in 152 articles, and only ten met the inclusion criteria: nine clinical articles and one in vitro study. One of the ten eligible articles was excluded. **Conclusions**: Fresh frozen rib cartilage proved to be a viable alternative to autologous rib grafts and irradiated homologous rib graft. Despite the higher costs, FFRG can provide a sufficient amount of tissue for grafting avoiding donor site complications and reducing the operative time and proved to have more chondrocytes and to be less prone to resorption compared to irradiated rib.

## 1. Introduction

Rhinoplasty is considered the principal cosmetic procedure in aesthetic facial plastic surgery accounting for more than 325,000 procedures in the US for the year 2020, according to the American Society of Plastic Surgery (ASPS) [1]. Patients’ main complaints are usually deformities and asymmetries that can be congenital or acquired (post-traumatic or iatrogenic). Moreover, according to the literature, 5–15% of patients undergo a revision rhinoplasty due to major complaint-s about the tip of the nose and the dorsum [2,3]. These are common drawbacks of aggressive resection leading, together with a lack of stabilization, to a loss of the support of nasal tissues. As a matter of fact, the insufficient support is reflected in the down-rotation of the tip, the loss of projection, the insufficient definition of the tip, polly beak deformities, and in-patent nasal valves. Revision surgery is burdened by three main issues: altered osteocartilaginous structure and a stiff and thick soft tissue envelope that needs stronger support. As a result, a rib graft may be needed to reconstruct the cartilaginous structure, depending on the entity of the over-resection and the elastic tension exerted by the soft tissue envelope. For many years, autologous rib cartilage represented the best solution to face complex reconstruction. Despite its advantages, it is not free of complications (infections, persistent pain, seroma and pneumothorax) and for this reason, alternative solutions were introduced in the latter years, such as alloplastic implants and irradiated homologous rib. The last innovation is the fresh frozen homologous (FFRG) rib that showed promising results in the substitution of autologous rib. This review aims to explore recent advances in structural rhinoplasty grafting with FFRG exposing all the available data in the literature.

## 2. Materials and Methods

In December 2023, literature research was conducted on PubMed, EMBASE, Medline and the Cochrane Library for all studies published from inception until December 2023. The searched keywords here combinations of “Rhinoplasty” and “Cartilage Graft”, “Allograft” or “Fresh Frozen Cartilage” in order to include all the available articles. The inclusion criteria were as follows: the use of FFRG as the main solution to correct residual nasal deformity; clinical and in vitro studies; exclusively English-written articles. Exclusion criteria were as follows: studies involving primary rhinoplasty without grafting, rhinoplasty with autologous grafts, irradiated homologous costal cartilage, non-surgical techniques and other alloplastic material. All the eligible articles were read by the authors and, if eligibility disagreements arose, a discussion between the authors solved them.

## 3. Results

**Figure 1:**
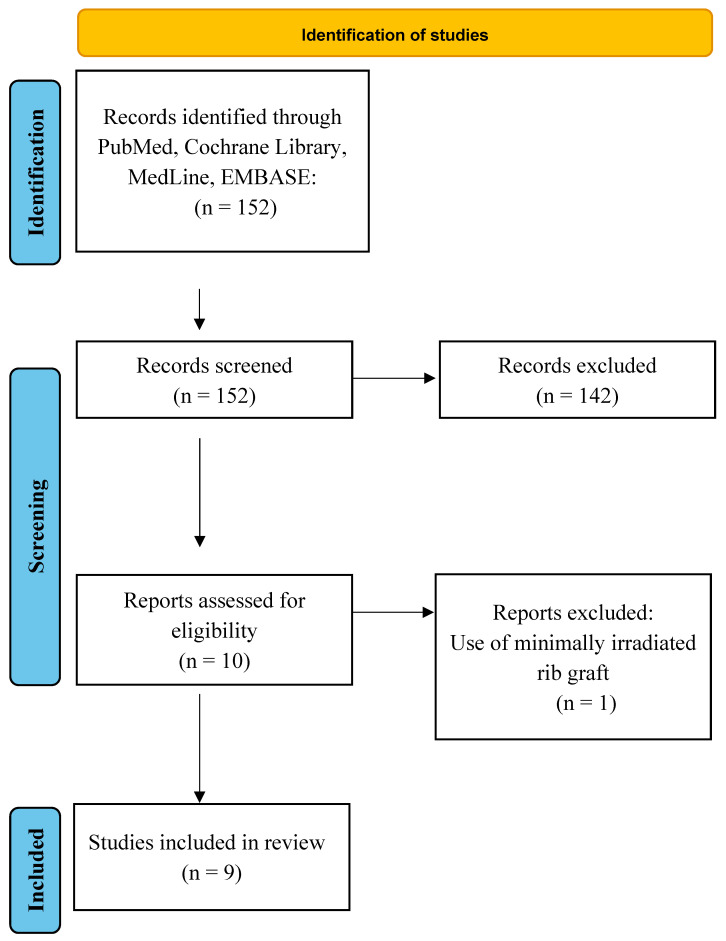
Details of the review search process.

The research resulted in 152 articles and only ten met the inclusion criteria: nine clinical articles and one in vitro study. One of the nine eligible clinical articles was excluded because the surgical treatment implied non- and minimally irradiated homologous costal cartilage without specifying the cases in which FFRG was used. (Figure 1) Out of the remaining eight clinical articles, six presented samples with less than 50 patients each, the smallest pool being of 5 patients. Besides these, two articles present bigger samples, with more than 200 patients. Moreover, it is noteworthy that four of these articles were written by the same group of authors. For this reason, some of the samples presented were overlapping. Given the novelty of the technique, it is reasonable that there is such a paucity of literature on this topic. Indeed, the included articles were published between 2019 and 2023, with the latest articles published in fall 2023.

### Clinical Findings

Rohrich et al. (2020) conducted an in vitro study to explore the warping characteristics of fresh frozen rib cartilage specimens related to different ages of the donor (20–35 y.o., 36–50 y.o. and >50 y.o.), storage temperatures (0 °C, 1.6 °C and 24 °C) and methods of use (cephalocaudal, oppositional or laminated orientation). The results of this study showed that fresh frozen cartilage is less subject to warping tendency if the donor was older than 36 years, the storage temperature was lower than 1 °C and the graft was harvested with oppositional orientation. The older age of the donor goes hand in hand with the calcification of the rib with consequent increased stiffness and decreased resorption. Younger cadavers showed more whitish rib cartilage that reflects a minor calcification being more prone to warping. The storage temperature is also crucial to reduce the warping phenomenon of the graft, less when stored frozen [4]. In 2020, Rohrich et al. showed no cases of resorption in 50 rhinoplasties with FFRG and only one case of infection related to the graft. Moreover, FFRG showed structural support and an ease of carving comparable to the irradiated rib but a lower warping tendency. When compared to autologous rib, FFRG showed less structural support, a similar warping tendency, easier carving and shorter operative times [5].

Mohan et al. presented a sample of 50 patients, operated on between 2014 and 2017 for revision rhinoplasty with FFRG and followed up with for a mean period of 3.5 months. The patients of this study underwent an average of two previous surgical treatments. The homologous grafts were used for dorsal onlay graft, spreader graft, columellar struts, infratip graft, septal extension graft and alar contour graft. The author showed only one case of postoperative infection treated with surgical debridement and antibiotics and no warping or resorption phenomena in this cohort. This study underlines the clinical importance of the age of the donor when using FFRG. FFRG proved to be useful to give support or to improve the contour but not in both contemporary. For this reason, the authors suggest using whiter grafts from younger donors for tip and alar contour grafts because they are softer and more pliable. More yellowish grafts from older donors are best suited for structural grafts like septal extension graft or the columellar strut because it is stiffer [6].

Wan et al. argued that the availability of septal cartilage in primary rhinoplasty in Asian patients may be inadequate. For this reason, they adopted FFRG on five primary rhinoplasties in Asian female patients resulting in good outcomes at the 1 year follow-up according to FACE-Q Satisfaction With Nose and Satisfaction With Nostrils scores with only one complaint about the surgical scar [7].

Milkovich et al. collected a cohort of 21 patients treated between 2019 and 2022 for primary or revision rhinoplasty (11 and 10, respectively) operated by a single surgeon using FFRG for septal extension, extended spreader, alar contour grafts and columellar struts mainly. The mean follow-up time was 15 months. Nineteen patients reported to be very satisfied with the final result, and two patients were satisfied but required a minor revision of the nose. No infection and no evidence of warping or deformities were observed. One patient experienced a resorption of the graft that was modified to be used as diced cartilage [8]. These studies share limitations like the small sample size and a short follow-up period but overall indicate a good outcome of FFRG use.

Recently, Rohrich et al. presented the largest case series of 226 patients treated for revision rhinoplasty over a period of nine years (2011–2020) and with a mean follow-up period of 12 months (from a minimum of 6 months to a maximum of 8 years). More than half of the patients (54%) underwent one prior intervention; 4% of the patients underwent four or more previous rhinoplasties. FFRGs were employed mainly for alar contour, septal extension grafts and columellar struts (49%, 40% and 23%, respectively). This study presented an infection rate of 2.7%, treated with medical therapy in most of the cases. One patient needed surgical intervention to explant the FFRG because of the infection. Four percent of patients experienced a mild erythema of the tip, self-limited in three weeks, that may have been caused by an immunologic reaction to the graft. The author observed a warping incidence of 2.7% and they did not observe any case of displacement or extrusion of the graft. However, the resorption rate was not routinely investigated by the author [9]. 

Novak et al. investigated the possible application of FFRG in the reshaping of the tip in primary rhinoplasty underlining the importance of considering nasal skin thickness, deviation or asymmetry of the dorsal aesthetic lines, C-shaped or reverse C-shaped nasal deformity, nasal tip shape, projection, rotation and deviation and a reported history of nasal trauma. All these aspects concur in the decision to use rib cartilage graft. The study was conducted on a sample of 30 patients (6 males and 24 females) operated by the same surgeon using fresh frozen allograft rib cartilage. All patients were followed up for at least 6 months. FFRG was used to carve out septal extension grafts for all patients and dorsal onlay graft for six of them. Due to the obvious difference in thickness between septal cartilage and FFRG, the author highlighted the need to taper the edges of the graft to decrease the palpability and the stiffness of the tip. Moreover, the pool of patients was sorted based on ethnicity since, especially in Asian and African American patients, this influences the thickness of the skin and consequently the projection of the nasal tip and the support needed to achieve the desired outcome [10]. 

In 2023, Wan et al. presented the result of the only prospective clinical trial in the literature to date, taking into account a pool of 50 patients, divided in two groups of 25 patients each between trial (FFRG) and control (autologous rib graft), that were treated between March 2017 and October 2020. The patients included in the study had to be nonsmokers and healthy males and females between 18 and 85 years old, undergoing cosmetic and/or reconstructive rhinoplasty that would have required grafts. All patients were documented with clinical pictures 6 months and 1 year after surgery in order to retrieve the objective assessment of the outcome via the measurements of the changing of the deviation angle, nasofrontal angle, total facial convexity, nasofacial angle and nasolabial angle. A subjective assessment of the outcome was performed through the FACE-Q questionnaire completed by the patients preoperatively and at 1 week, 6 weeks, 12 weeks, 6 months and 12 months postoperatively. The objective assessment showed greater changes in the deviation angle, nasofrontal angle, total facial convexity and nasolabial angle in the control group, while changes in the nasofacial angle were greater in the FFRG group. The author of the trial presented a general increase in subjective satisfaction after surgery in both groups, without a statistically significant difference between them. Among the 50 patients, a total of 16 patients experienced complications with a greater incidence in the control group (10 cases) than the FFRG group (6 cases). Complications in the FFRG group listed two cases of warping (three cases in the control group) as well as two cases of resorption (the same in control group) and two pathological healings of the scar. In the control group, one patient experienced an infection of the graft. No infections were reported in the FFRG group. Nonetheless, even with the high costs of shipping and storing frozen cartilage, the total surgery expense was still lower than using autologous costal cartilage. Additionally, there was a significant reduction in both surgical fees and the need for narcotic medications [11]. 

Hanna et al. presented the biggest case review of rhinoplasties with FFRG so far. The author reviewed the outcomes of interventions on 282 patients, mainly female (90%), treated between March 2018 and December 2021, followed up with for at least 12 months. Both primary (14.2%) and revision rhinoplasty (85.8%) were included in the pool of patients. In the study, 2.1% of patients experienced infections that were completely solved with empiric antibiotics. Six patients needed revision surgery during which the FFRG was inspected showing no significant warping, resorption or displacement. On the contrary, the graft appeared to be well integrated with the surrounding tissue. Based on the experience matured during this period of time, the author presented a few considerations to improve the quality of the outcome when using FFRG. First of all, the thawing of the graft should last at least one hour prior to the usage. A frozen graft appears perfectly straight, while an accurate thawing reveals any inherent warping of the rib that can be thrown away once carved. Moreover, the author prefers to use more yellowish FFRG (from older donors) to improve the support of the cartilage structure thanks to the increased calcification of the rib. As a matter of fact, FFRGs were adopted mostly to fashion spreader and columellar strut grafts avoiding using them for tip grafts and dorsal onlay grafts because they are too rigid and firm. Nonetheless, it is highlighted that FFRG does not respond to scoring like the septal cartilage would. For this reason, it is not possible to use this technique to straighten the rib graft more. To continue, FFRG appeared to be more prone to fracture when sutured, compared to the septal cartilage [12]. All the results are summarized in Table 1, Table 2 and Table 3.

## 4. Discussion

With the aim of reducing the mass and volume of the nose, techniques emphasizing the removal of bone, cartilage and fat proliferated. The first consequence of this increasing trend was a proportional increase in patients suffering from consequences due to excessive removal. Despite an improved comprehension of the long-term impacts of excessively aggressive techniques, rhinoplasty remains among the most intricate and demanding surgical procedures, requiring great experience not only due to technicalities but also for the possible drawbacks influencing patients’ emotional, respiratory, biobehavioral and immunologic factors. About 20% of postoperative nasal deformities may require additional surgical intervention, varying between studies. Conservative estimates suggest that around 26% of secondary rhinoplasty consultations are attributed to over-resection, resulting in patient dissatisfaction [13]. Reducing septal height or strength through over-resection or aggressive manipulation can result in a saddle-nose deformity or deviations along the dorsal and caudal septum. Removing too much cartilage from the upper nose can cause the middle part to narrow excessively, resulting in the collapse of the internal nasal valve, or it can lead to an inverted-V shape after reducing a nasal hump. The lower third of the nose is the most challenging step for a successful rhinoplasty. The projection of the tip of the nose depends on preserving a specific cartilage complex together with the quality and thickness of the skin. All these represent crucial aspects during the planning phase of the surgery. Excessive removal can lead to the collapse of the external nasal valve, causing a pinched look of the nasal tip, the retraction of the alar and a loss of tip definition. Moreover, excessive reduction in the alar rim may result in the loss of the natural flare of the alar and overly narrow nostrils. Similarly, overly aggressive cephalic trimming towards the upper part of the lower alar cartilage can cause the columella to be excessively visible. These are just a few of the many aspects that have to be taken into consideration while planning a revision surgery that needs to fulfill a patient’s aesthetical expectations and functional aspects of a healthy nose (the patency of internal and external nasal valves and the correct anatomy of inferior turbinates). An in-depth study of every single case is mandatory to plan an adequate treatment and it should include: clinical examination (an analysis of nasal skin, deviation, airway obstruction, dorsal width, tip deviation, tip shape, alar retraction/notching/flaring, alar base width, nasal length, dorsal convexity/concavity, tip projection/rotation and columellar show/deviation), pictures and Cone Beam CT to further investigate the underlying structure [14]. Therefore, secondary surgery is burdened by three main issues: an altered osteocartilaginous structure, a stiff and thick soft tissue envelope that needs stronger support and, last but not least, previous surgery details are often missing or unknown before surgery. These three aspects get worse after every surgical procedure because, besides the biological response to repeated surgical trauma, the available stocks of cartilage for grafts run lower and lower. Moreover, particular attention should be dedicated to those cases that present intraoperatively traumatized or congenitally weak/deformed lower lateral cartilages, so that the need for a rib graft cannot be decided preoperatively despite the quality of the clinical assessment and surgical planning. 

### 4.1. Autograft and Allograft to Restore the Lost Cartilaginous Structure

Grafts and implants help resist both static forces like gravity and aging, as well as dynamic forces from tissue contraction, scarring, the activity of muscles and normal breathing pressure changes. If septal and auricular cartilage may be a valid workhorse in primary or mild secondary rhinoplasty, they are insufficient in most severe cases in which previous surgery over-resected the nasal skeleton (for example, patients with significant deformities needing a nasal dorsum increase beyond 4 mm). It was recently estimated that in 13% of all cases (more often in revision surgery compared to primary), extra nasal cartilage is required [15]. Furthermore, harvesting septal cartilage graft might not be an option when the patient has undergone septoplasty or there is a post-traumatic or drug abuse-related septal perforation. In all these cases, autologous rib cartilage represents the gold standard guaranteeing plenty of material for grafting. Autologous rib is highly biocompatible, and it is characterized by the low rate of resorption and infections. Despite this main advantage, it is not free of complications. Donor site complications are relatively uncommon and may include infections, persistent pain, seroma, chest wall deformity and the pathological healing of the wound. Pneumothorax is reported to occur more than 20% of the time in costal cartilage graft harvests for microtia reconstruction but is less common with smaller rib segments used in rhinoplasty (0.1–2%) [16,17,18]. Moreover, complications at the recipient site can involve problems with graft sizing, position, mobility and warping. Another disadvantage of harvesting an autologous rib graft is the necessity of general anesthesia and increased surgical costs. Potential drawbacks, like donor site morbidity, longer operating times and the need for more graft material, have led surgeons to consider synthetic materials. Despite obvious advantages of these medical devices (highly availability, no need for a second surgical site and lower cost), they are burdened by higher rates of mobilization, infection and extrusion, especially in areas under tension or in patients with thin skin. In the last few years, we assisted the introduction of homologous solutions that could represent a viable alternative: irradiated rib cartilage and, more recently, fresh frozen rib cartilage. Complication rates for cadaveric cartilage in the literature vary from 3.25% to 45%. In a recent study by Vila et al., which included over 1000 patients, there was no significant difference in complications (warping, resorption, contour irregularity, infection and the need for revision) between autologous grafts, irradiated homologous costal cartilage grafts and Tutoplast (RTI Surgical Holdings, Inc., Alachua, FL, USA) cartilage grafts for dorsal augmentation rhinoplasty [19]. Lately, survey research was conducted inviting expert surgeons (over 50 rhinoplasties per year) to answer a questionnaire about the possible application of cadaver rib grafts in rhinoplasty. The majority (76.8%) of the interviewed surgeons affirmed the use of cadaver rib grafts. Surgeon preference was the main reason for choosing cadavers over autografts (71.3%), suggesting that facial plastic surgeons are more comfortable using cadaver allografts, when possible, compared to autografts. The main reasons that affect the use of cadaveric rib grafts were concerns about patient health, potential complications, the annual number of rhinoplasties performed and the cost of cadaveric ribs. It is also possible to assume another practical factor: while performing 50 rhinoplasties per year, cadaveric grafts play a crucial part in saving time and increased surgeon comfort. Shorter operating times were also reflected in safer surgical procedures in fragile patients or subjects with comorbidities. This was confirmed in the survey where an allograft was preferred over an autologous graft depending on the patient’s medical conditions (61.8%) and shorter procedure or stay times (57.9%) and, more in general, all those scenarios where the concern for a longer procedure or an additional surgical site is greater than the risk of allograft complications. On the other hand, 10% of interviewed surgeons did not use cadaver grafts due to concerns about infection and warping, and 28% were worried about the rate of resorption. These allografts are available in different sizes helping to reduce the operative time spent trimming the graft and the graft waste, and a recent meta-analysis showed no significant differences between autologous and homologous grafts when used for dorsal augmentation [4,19,20]. On the other hand, Wee et al. proved that irradiation caused a decrease in chondrocyte availability, compromising the graft integrity and increasing the incidence of infection and resorption (as high as 31%) compared to autologous rib grafts (3%), despite the decreased dose of gamma radiations (from 50,000 Gy to 25,000 Gy) [21,22]. Contrarily to the available studies about irradiated grafts, FFRG is an emerging solution that has the potential to avoid issues related to irradiation. 

### 4.2. Harvesting Technique

To date, fresh frozen cartilage is made by the Musculoskeletal Tissue Foundation (Edison, N.J.), and it is harvested from donors younger than 55 years, who tested negative for HIV, HBV and HCV and with no sepsis or malignancies. According to data, less than 2% of available donors get accepted for further tissue processing. The cartilage is harvested from the seventh to the ninth rib, debrided of the soft tissue envelope and trimmed to size. The specimen is then rinsed with a surfactant to remove blood and cellular components, soaked and decontaminated in antibiotic solution. The final product is then stored sterile under frozen conditions (−40 °C to −80 °C), ready to be shipped in dry ice. In the operating room, the FFRG is unfrosted and made ready to use [23]. FFRG is available in sheets of fourteen different sizes or a rib segment, available in two shapes. The costs for FFRG start from a base of USD 200 to a maximum of USD 800. 

### 4.3. Future Possibilities beyond Allogenic Rib Graft

The high interest in rhinoplasty, and the constant increase in the number of procedures performed per year, proportionally increases the risk of unfavorable outcomes, especially when the surgery is performed by unexperienced surgeons. This would lead to an always increasing number of patients looking for revision surgery and, the more intervention they undergo, the worse the scenario would be that the next surgeon would have to face. The need for the structural support of the nose demands more grafting techniques. Nowadays, many efforts are made by industries to fulfill the need of allografts usable in substitution for rib cartilage. For example, Osiris Therapeutics, Inc. (Columbia, MD, USA) introduced Osiris Cartiform made out of hyaline osteochondral allograft containing growth factors, chondrocytes and extracellular matrix proteins, and it is employed in articular reconstructions with apparent benefits [24]. Application in the nose district still has to be studied. Currently, we are assisting an ongoing development in the field of tissue engineering that is proving to be able to create bioengineered tissues to replace atrophic or pathologic cartilage for articular cartilage, knee meniscus and temporomandibular joint disc applications. This success in engineering cartilage is motivating its application for nasal cartilage tissue engineering. Currently, the idea is to create a scaffold-free tissue engineering approach providing easily accessible, abundant and robust cartilage [25].

### 4.4. Limitations

Despite the evidence, this review has some limitations. First of all, the exiguity of articles available in the literature gives an idea of what this device can be in surgical practice, but the results are still not enough compared with more investigated autologous rib grafting. The data available in the literature were not homogenously collected resulting in the partial loss of details and the impossibility of a true comparison between studies. This last limitation is even stronger taking into account the two biggest case series, counting more than 200 patients each, in which the inhomogeneous data collection between them affects the comparison. Nonetheless, most of the articles available in the literature were written by the same research group creating an overlap of the patients treated between the studies and another that weakens the scientific resonance of the data. Finally, the available data do not include mean long-term results greater than 12 months and therefore is difficult to assess the long term stability and complications like warping or graft resorption. 

## 5. Conclusions

The advent of FFRG can represent a great shift for revision rhinoplasty surgery. This review showed that FFRGs have similar outcomes to autologous ribs and less complications than radiated homologous ribs. Nowadays, autologous rib graft represents the gold standard for its biocompatibility and low rates of infection. However, FFRG can be a valid alternative, avoiding donor site morbidity and the risk of pneumothorax during harvesting. The biological behavior shown in these clinical reports together with the low rate of local complications may justify the increased costs of the surgery. Nonetheless, FFRG is readily available off-the-shelf shortening operative time.

## Figures and Tables

**Table 1:** Population epidemiology.

Author	Population	Male	Female	Mean Age (Years)	Mean Follow-Up (Months)	Previous Rhinoplasty
Rohrich (2020) [5]	50 patients	Not specified	Not specified	Not specified	Not specified	Not specified
Mohan (2019) [6]	50 patients	12	38	40 (range 21–70)	3.35	Not specified
Wan (2021) [7]	5 patients	0	5	Not specified	14.2	Not specified
Milkovich (2022) [8]	21 patients	4	17	39 (range 27–58)	15	10 out of 21
Rohrich (2022) [9]	226 patients	41	185	40.6 (range 19–74)	12.2	104 out of 226
Novak (2023) [10]	30 patients	6	24	Not specified	Minimum 6 months	0 out of 30
Wan (2023) [11]	25 patients	5	20	38.8 (range 20–83)	14.8	4 out of 25
Hanna (2023) [12]	282 patients	27	225	35.8 (range 15–68)	20.3	242

**Table 2:** Complication rates.

Author	Complications	Warping	Resorption	Infection	Extrusion/Displacement	Other
Rohrich (2020) [5]	1 out of 50	Not specified	0	1 out of 50	Not specified	Not specified
Mohan (2019) [6]	1 out of 50	0	0	1 out of 50	0	0
Wan (2021) [7]	1 out of 5	0	0	0	0	1 pathological scarring
Milkovich (2022) [8]	1 out of 21	0	1	0	0	0
Rohrich (2022) [9]	21 out of 226	6	Not specified	6	0	9 tip erythema
Wan (2023) [11]	6 out of 25	2	2	0	0	2 pathological scarring
Hanna (2023) [12]	12 out of 282	0	0	6 out of 282	0	0

**Table 3:** Graft types carved from FFRG.

Author	Septal Extension	Columellar Strut	Alar Contour	Dorsal Onlay	Spreader	Infratip	Diced Cartilage
Rohrich (2020) [5]	Not specified	Not specified	Not specified	Not specified	Not specified	Not specified	Not specified
Mohan (2019) [6]	6%	28%	88%	30%	16%	16%	0%
Wan (2021) [7]	Not specified	Not specified	Not specified	Not specified	Not specified	Not specified	Not specified
Milkovich (2022) [8]	61.9%	42.8%	76.1%	9.5%	47.6%	9.5%	4.8%
Rohrich (2022) [9]	40%	23%	49%	12%	Not specified	Not specified	Not specified
Novak (2023) [10]	100%	0%	0%	16.7%	0%	0%	0%
Wan (2023) [11]	13%	10%	5%	10%	45%	17%	Not specified
Hanna (2023) [12]	Not specified	Not specified	Not specified	Not specified	Not specified	Not specified	Not specified

## Data Availability

No new data were created or analyzed in this study. Data sharing is not applicable to this article.
